# Failure to activate the IFN-β promoter by a paramyxovirus lacking an interferon antagonist^☆^

**DOI:** 10.1016/j.virol.2011.03.027

**Published:** 2011-06-20

**Authors:** M.J. Killip, D.F. Young, C.S. Ross, S. Chen, S. Goodbourn, R.E. Randall

**Affiliations:** a School of Biology, Centre for Biomolecular Sciences, BMS Building, North Haugh, University of St. Andrews, St. Andrews, Fife KY16 9ST, UK; b Division of Biomedical Sciences, St. George's University of London, London SW17 0RE, UK

**Keywords:** Interferon, Interferon antagonist, Paramyxovirus, PIV5, Defective viruses

## Abstract

It is generally thought that pathogen-associated molecular patterns (PAMPs) responsible for triggering interferon (IFN) induction are produced during virus replication and, to limit the activation of the IFN response by these PAMPs, viruses encode antagonists of IFN induction. Here we have studied the induction of IFN by parainfluenza virus type 5 (PIV5) at the single-cell level, using a cell line expressing GFP under the control of the IFN-β promoter. We demonstrate that a recombinant PIV5 (termed PIV5-VΔC) that lacks a functional V protein (the viral IFN antagonist) does not activate the IFN-β promoter in the majority of infected cells. We conclude that viral PAMPs capable of activating the IFN induction cascade are not produced or exposed during the normal replication cycle of PIV5, and suggest instead that defective viruses are primarily responsible for inducing IFN during PIV5 infection in this system.

## Introduction

The interferon (IFN) response is an extremely powerful antiviral defence mechanism that can control early phases of virus replication within the host. To facilitate replication and spread, most (if not all) viruses therefore employ mechanisms to at least partially circumvent the IFN response (reviewed by Randall and Goodbourn, 2008). Nevertheless, the ability of viruses to circumvent the IFN system is not absolute and the IFN response remains critical in slowing the progress of virus infections, thereby buying time for the development of an adaptive immune response.

The current model of IFN induction holds that viruses generate molecules (referred to as pathogen-associated molecular patterns, or PAMPs) during their normal replication cycle that are not found in uninfected cells. These PAMPs are specifically recognised by cellular pathogen-recognition receptors (PRRs) that trigger downstream IFN induction pathways (Takeuchi and Akira, 2009; Wilkins and Gale, 2010). RIG-I and MDA-5 are two such cytoplasmic PRRs; RIG-I recognises short, 5′-triphosphorylated double-stranded RNA, whilst MDA-5 recognises longer molecules of dsRNA which need not be 5′-triphosphorylated (Kato et al., 2008; Lu et al., 2010; Schlee et al., 2009; Schmidt et al., 2009). Following binding of their appropriate ligands, RIG-I and MDA-5 homo-oligomerise to reveal their CARD domains, thereby facilitating their interaction with the CARDIF/MAVS/IPS1/VISA adaptor protein. As a consequence of this interaction, the IFN induction signal is transmitted to a number of downstream kinases, including IKKα/β and TBK1/IKKε, resulting in activation of the transcription factors NF-κB and IRF3, respectively. These transcription factors, together with ATF-2/cJUN, are required for activation of the IFN-β promoter, which ultimately results in the secretion of IFN-β from infected cells. Secreted IFN binds to cell surface receptors and activates intracellular signalling cascades to promote an antiviral state by upregulating the expression of many interferon-stimulated genes (ISGs) (Platanias, 2005; Randall and Goodbourn, 2008).

Viruses use a variety of strategies to limit IFN production. These include encoding products that interfere with specific cellular components of the IFN induction cascade, or which block cellular transcription and/or protein synthesis (Haller et al., 2006; Randall and Goodbourn, 2008). The paramyxovirus parainfluenza virus type 5 (PIV5) encodes the V protein, which is well characterised as an IFN antagonist. V specifically binds to MDA-5, precluding it from binding dsRNA and preventing its downstream signalling functions (Andrejeva et al., 2004; Childs et al., 2007, 2009), and has also been reported to act as a competitive inhibitor of TBK-1, the kinase that phosphorylates and activates IRF3 (Lu et al., 2008). In addition to inhibiting IFN induction, the V protein also acts to block IFN signalling by targeting STAT1 for proteasome-mediated degradation (Didcock et al., 1999; Precious et al., 2007). Thus, a recombinant virus, termed PIV5-VΔC, which makes a non-functional C-terminally truncated V fragment, is extremely sensitive to the IFN system, being unable to either block IFN signalling or limit IFN production (He et al., 2002; Poole et al., 2002).

To follow activation of the IFN response in individual cells, we recently generated a cell line, which reports faithfully the induction of IFN-β at a single-cell level (Chen et al., 2010). Using this cell line, we showed that there is heterocellular induction of IFN by negative strand RNA viruses. This phenomenon has been observed several times previously but was assumed to be a property of the host cell (Apostolou and Thanos, 2008; Enoch et al., 1986; Hu et al., 2007; Senger et al., 2000; Zawatzky et al., 1985). We demonstrated instead that, in our system, it was a feature of the infecting virus as opposed to an intrinsic property of the cells. Here we demonstrate that this heterocellular activation of the IFN-β promoter also occurs for PIV5 lacking an IFN antagonist, as PIV5-VΔC does not activate the IFN-β promoter in the majority of infected cells. On the basis of our results, we propose that PAMPs that can activate the IFN response are not produced or exposed during the normal transcription and replication processes of PIV5.

## Results

### PIV5-VΔC induces GFP expression in A549/pr(IFN-β).GFP cells

**Fig. 1:**
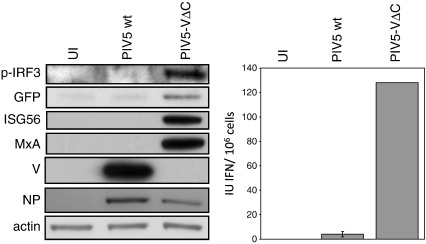
Activation of the IFN response by PIV5-VΔC in A549/pr(IFN-β).GFP reporter cells. A549/pr(IFN-β).GFP cells were infected with PIV5 wt or PIV5-VΔC at an MOI of 5 PFU/cell. Uninfected (UI) cells were included as a negative control. Twenty-four hours post-infection, culture media were harvested and cell lysates were prepared. Lysates were subjected to immunoblotting with antibodies specific to phosphorylated (active) IRF3, GFP, ISG56, MxA, PIV5 V (C-terminus), actin and viral NP (left panel). IFN present in culture media was estimated by a CPE-reduction bio-assay and converted to IU IFN/10^6^ cells using an IFN-α standard. The mean IFN produced in three independent experiments is plotted (right panel) and error bars represent the SD for each triplicate.

We have previously generated and characterised a reporter cell line, A549/pr(IFN-β).GFP, in which GFP is expressed under the control of the IFN-β promoter (Chen et al., 2010). Here we have used these cells to study the process of IFN induction by a recombinant PIV5 that lacks a functional IFN antagonist (PIV5-VΔC). PIV5-VΔC encodes a C-terminally truncated version of the V protein which cannot interact with MDA-5 or target STAT1 for proteasome-mediated degradation and is consequently impaired in its ability to inhibit IFN induction and block IFN signalling in infected cells (He et al., 2002; Poole et al., 2002). As expected, IFN was induced in A549/pr(IFN-β).GFP cells infected with PIV5-VΔC, as indicated by an increase in IRF3 phosphorylation, IFN-α/β secretion and expression of GFP relative to PIV5 wt-infected cells (Fig. 1). Furthermore, increased expression of the ISG products ISG56 and MxA was observed in PIV5-VΔC-infected cells, indicating that this virus was unable to block IFN signalling. These data show, as has been shown previously, that truncation of the PIV5 V protein leads to an increase in IFN production in infected cells (He et al., 2002; Poole et al., 2002). This activation of the IFN-β promoter is accompanied by an increase in GFP expression in our reporter cells, indicating that the A549/pr(IFN-β).GFP cell line is valid for use in studying activation of the IFN response by PIV5-VΔC.

### Heterogeneity in IFN-β promoter activity in PIV5-VΔC-infected cells

**Fig. 2:**
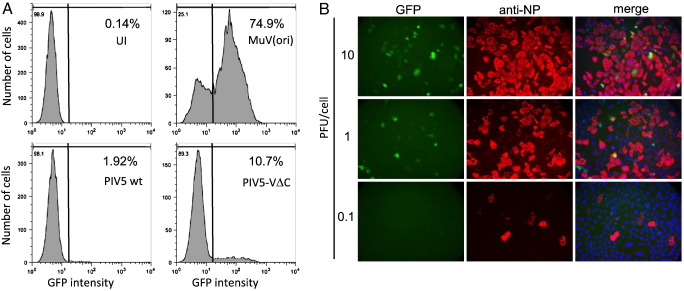
The IFN-β promoter is activated in only a subset of PIV5-VΔC-infected cells. (A) A549/pr(IFN-β).GFP cells were infected with PIV5 wt, PIV5-VΔC or MuV(ori) (as a positive control) at 5 PFU/cell. Uninfected (UI) cells were included as a negative control. Sixteen hours post-infection, cells were trypsinised, fixed and subjected to flow cytometry analysis to determine GFP expression. The percentage of cells considered to be GFP-positive (based on the line gate indicated) is given in the top right hand of each panel. (B) A549/pr(IFN-β).GFP cells were infected with PIV5-VΔC at 10, 1 or 0.1 PFU/cell. Sixteen hours post-infection, cells were fixed and immunostained for viral NP expression. GFP (green) and NP (red) were visualised by fluorescence microscopy. The presence of the nuclei (blue) in the merged images was visualised by DAPI staining.

Since it is generally thought that PAMPs are produced during normal virus replication and the V protein functions to block activation of the IFN response by these replication products, we reasoned that the detectable markers of IFN induction seen in the cell population experiments described above (Fig. 1) would be reflected in the activation of the IFN-β promoter by PIV5-VΔC in every infected cell. Flow cytometry analysis of A549/pr(IFN-β).GFP cells infected with wild-type PIV5 or with PIV5-VΔC was used to monitor GFP expression in individual cells (Fig. 2A). As expected, and consistent with PIV5 wt being a poor inducer of IFN expression, GFP was detectable in only a small percentage (< 2%) of cells infected with PIV5 wt at high multiplicity. However, surprisingly, in cells infected with PIV5-VΔC, although there was an increase in the total number of GFP-positive cells compared to wt virus infection, only a minority (10.7%) of infected cells were positive for GFP; the majority of cells remained GFP-negative. These results were confirmed by immunofluorescence, which clearly showed that the majority of PIV5-VΔC-infected cells were negative for GFP (Fig. 2B). The low numbers of GFP-positive cells seen with these viruses were unlikely to be due to variation in the ability of individual A549/pr(IFN-β).GFP cells to respond to IFN inducers since ~ 75% of cells expressed GFP when infected with MuV(ori), a stock of mumps virus known to be a good inducer of IFN due to the presence of defective viruses (Fig. 2A; Chen et al., 2010). It is thus clear from these data that in this system, the IFN-β promoter is active in only a small subset of cells infected with PIV5-VΔC and it is likely that these cells are responsible for the IFN secreted by PIV5-VΔC-infected cell populations.

### Heterogeneity in IFN-β promoter activity in developing plaques of PIV5-VΔC

**Fig. 3:**
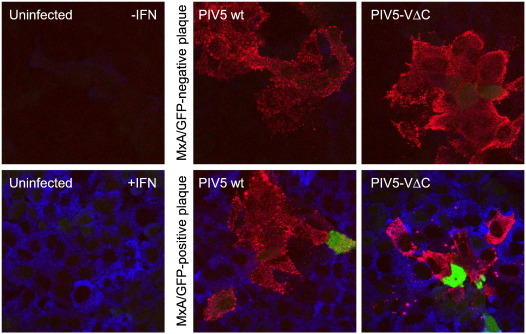
Heterocellular activation of the IFN-β promoter in PIV5-VΔC developing plaques. A549/pr(IFN-β).GFP cells were infected with either PIV5 wt or PIV5-VΔC to obtain discrete virus plaques. Two days post-infection, cells were fixed, permeabilised and immunostained for viral NP and cellular MxA. MxA is upregulated by IFN and is therefore indicative of IFN response activation. Uninfected cells were treated with IFN-α as a positive control for MxA expression. GFP (green), NP (red) and MxA (blue) were visualised by fluorescence microscopy.

We next examined whether the heterocellular activation of the IFN-β promoter seen in response to PIV5-VΔC infection is seen during the development of virus plaques. PIV5-VΔC is extremely sensitive to the effects of IFN, and so only forms small plaques in IFN-competent A549/pr(IFN-β).GFP cells; nevertheless, plaque development can be followed at early times p.i., before enough IFN is produced to prevent further plaque development. As seen for PIV5 wt (Fig. 3, middle panels; Chen et al., 2010), the IFN-β promoter was only activated in a minority of cells within developing PIV5-VΔC plaques at 2 days post-infection (Fig. 2, right panels). As expected, the uninfected cells surrounding plaques containing a GFP-positive cell were positive for MxA expression, indicating activation of the IFN response. However, plaques could also be seen that contained no GFP-positive cells, indicating that the IFN-β promoter had not been activated within these developing plaques. Furthermore, an antiviral state had not been established in the uninfected cells surrounding these plaques (as demonstrated by a lack of MxA expression; Fig. 2, top right panel), indicating that no endogenous IFN-β had been secreted by any of the infected cells in the plaque. [It should be noted that as previously observed for PIV5 wt (Chen et al., 2010), all plaques had at least one GFP-positive cell in them by 3–4 days p.i. At this point, the further development of PIV5-VΔC plaques is prevented, in contrast to PIV5 wt plaques which continued to develop slowly due to the ability of the wt virus to dismantle the IFN-induced antiviral state (Carlos et al., 2009; Precious et al., 2007).] These results confirm that the IFN-β promoter is not activated in the majority of cells infected with PIV5-VΔC, demonstrating that PAMPs capable of inducing IFN are not generated during PIV5-VΔC transcription and replication processes.

### Induction of IFN by PIV5-VΔC correlates with the presence of DI viruses

For other negative strand RNA viruses including Sendai virus, mumps virus and VSV (Chen et al., 2010; Fuller and Marcus, 1980; Johnston, 1981; Marcus and Gaccione, 1989; Sekellick and Marcus, 1989; Strahle et al., 2006), it has been shown that virus stocks that are rich in defective interfering (DI) viruses are powerful activators of the IFN response. Since our data clearly demonstrate that PAMPs capable of activating the IFN response are not generated during normal PIV5-VΔC transcription and replication, we sought to determine whether DI viruses are primarily responsible for inducing IFN in PIV5-infected cells.

**Fig. 4:**
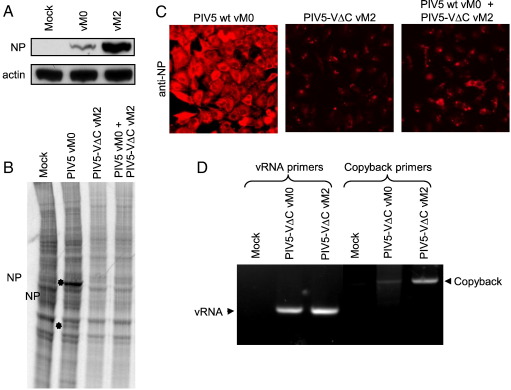
Generation of DI-rich preparations of PIV5-VΔC. (A) A549/pr(IFN-β).GFP cells were infected at 5 PFU/cell with PIV5-VΔC vM0 or vM2 stocks, or mock-infected, in the presence of cycloheximide (50 μg/ml). Two hours later, monolayers were harvested and analysed for input virus NP and actin by SDS-PAGE and immunoblotting. (B) A549/pr(IFN-β).GFP cells were infected at an MOI of 10 PFU/cell with PIV5 wt vM0, PIV5-VΔC vM2 or 50:50 mixtures of both of these viruses, or mock-infected. At 20 h p.i. the monolayers were radioactively labelled with [^35^S]methionine and the labelled polypeptides in total cell extracts were visualised by SDS-PAGE analysis and autoradiography. The positions of the viral NP and M proteins are highlighted with an *. (C) A549/pr(IFN-β).GFP cells were infected at an MOI of 10 PFU/cell with PIV5 wt vM0, PIV5-VΔC vM2 or 50:50 mixtures of both of these viruses. At 20 h p.i. cells were fixed and the distribution of NP was visualised by immunostaining and fluorescence microscopy. (D) Total RNA was prepared from either mock-infected 293 cells or 293 cells infected at an equivalent PFU/cell with either PIV5-VΔC vM0 or PIV5-VΔC vM2, and then subjected to RT-PCR as described in Materials and methods. Reverse transcription was performed with primer A or B, and then PCR was carried out with primer pairs B + C (vRNA primers) or A + C (copyback primers). Primers B and C are in opposing orientations and permit amplification of any PIV5 RNA generated by authentic replication. Primers A and C are both from the same strand and can only generate a PCR product if the template has switched strands. Products were analysed on a 1.2% agarose gel and the presence of vRNA and copyback molecules is indicated. The faint DNA fragment seen in the mock-infected sample with the copyback primers has a similar but distinct mobility to that seen with the copyback fragment and is non-specific.

DI virus-rich stocks of PIV5-VΔC were generated by sequentially passaging the virus at high multiplicity, a process known to increase the number of DI viruses in the virus population. Sequential high MOI passages are referred to as vM1, vM2, etc., after Von Magnus (1951) who first showed that high MOI passage of influenza virus leads to the generation of DI viruses. The presence of DI viruses in our vM2 stock could be demonstrated by examining the levels of input NP protein in cells infected with the vM0 and vM2 viruses at equal multiplicity in the presence of cycloheximide. Fig. 4A shows substantially more NP present in vM2-infected cells than for vM0, indicating an increase in the total number of input virus particles relative to the infectious titre. In addition, the vM2 virus preparation inhibited the replication and protein expression of PIV5 wt (Fig. 4B and C), a characteristic that has been used previously to estimate the numbers of DIs in a virus stock (Marcus et al., 2009).

In previous studies on the DI particles of paramyxoviruses it has been shown that IFN induction is associated with the presence of copyback genomes thought to be generated by template switching during replication (Strahle et al., 2006; Takaki et al., 2011). We therefore investigated whether these copyback genomes could be detected in our PIV5-VΔC stocks using RT-PCR on RNA extracted from infected cells and applying the primer strategy devised by Roux and Kolakofsky (Calain et al., 1992). In contrast to our ability to detect vRNA at equivalent levels in RNA samples from cells infected by either vM0 or vM2 stocks we observed a fragment amplified by the same-sense copyback primer pair that, although present in the vM0 sample, was more abundant in the vM2 sample (Fig. 4C). This fragment was recovered and shown by DNA sequencing to be derived from a template switching event in the RNA such that PIV5 nucleotide 14,043 is transposed next to nucleotide 15,023 in the opposite strand. This event would generate a predicted copyback DI particle of 1428 nt in length, with a perfect dsRNA stem of 223 bp, and this particle obeys the rule-of-six.

**Fig. 5:**
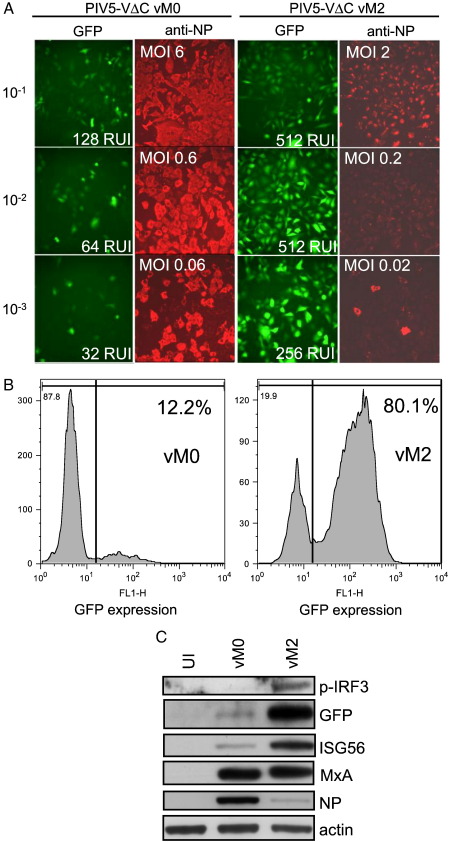
Induction of IFN by PIV5-VΔC correlates with the presence of DI viruses. (A) Equivalent dilutions of PIV5-VΔC were used to infect A549/pr(IFN-β).GFP cells. Cells were fixed at 16 h post-infection and immunostained for NP expression. GFP (green) and NP (red) were visualised by fluorescence microscopy. Culture media were collected from infected cells and subjected to a CPE-reduction bio-assay to estimate the IFN present. Relative units of IFN (RUI) produced and the MOI (PFU/cell) for each virus dilution are shown as insets in the GFP and anti-NP panels, respectively. (B) A549/pr(IFN-β).GFP cells were infected with PIV5-VΔC vM0 and vM2 preparations at 1–2 PFU/cell. At 16 h post-infection, cells were trypsinised, fixed and subjected to flow cytometry analysis to determine GFP expression. The percentage of cells considered to be GFP positive (based on the line gate indicated) is given in the top right hand of each panel. (C) Lysates of A549/pr(IFN-β).GFP cells infected with 5 PFU/cell of PIV5-VΔC vM0 or vM2 for 24 h were subjected to immunoblotting with antibodies specific to phosphorylated IRF3 (p-IRF3), GFP, ISG56, MxA, viral NP and actin. Uninfected (UI) cells were included as a negative control.

The induction of IFN and GFP expression increased dramatically when A549/pr(IFN-β).GFP cells were infected with this vM2 stock compared to our original (vM0) stock of PIV5-VΔC (Fig. 5A). Thus, following infection with PIV5-VΔC vM0 only a small proportion of cells were GFP-positive, while in striking contrast, PIV5-VΔC vM2 resulted in the majority of cells expressing GFP. This increase in GFP expression was consistent with a large increase in IFN secretion by infected cells (Fig. 5A). Differences in GFP expression were quantified by FACS analysis: following infection of the A549/pr(IFN-β).GFP cells with 1–2 PFU/cell, ~ 12% of cells were positive for GFP following infection with PIV5-VΔC vM0, while ~ 80% were positive following infection with PIV5-VΔC vM2 (Fig. 5B). PIV5-VΔC vM2-infected cell lysates also exhibited considerably more IRF3 phosphorylation than PIV5-VΔC vM0-infected cells (Fig. 5C). Interestingly, there was no difference in the level of MxA expression in the vM0- vs. vM2-infected cells, presumably because all the PIV5-VΔC-infected cells would have responded to any IFN produced due to the inability of PIV5-VΔC to block IFN signalling. In contrast the level of ISG56 was higher in the vM2-infected cells compared to the vM0-infected cells, because, in addition to being upregulated by IFN, its expression is also directly induced by activated IRF3 (Grandvaux et al., 2002). It is likely then that the majority of ISG56 induced in the vM2-infected cells had been induced by IRF3. It is also of note that the 10^−3^ dilution of vM2 virus (0.02 pfu/cell) induced significantly more IFN- and GFP-positive cells than the 10^−1^ dilution of the vM0 virus (6 pfu/cell). This demonstrates that the ability of the vM2 virus to induce IFN relative to vM0 was not due to either an increase in the amount of virus binding to the cell or an increase in the number of input virus genomes. Plaque purification of PIV5-VΔC from the DI-rich vM2 stock reduced its ability to induce IFN to that observed with our original non-defective stock, showing that it was the accumulation of DI viruses that was responsible for the increase in IFN induction, and not any selected genetic changes in the properties of the non-defective virus that may occur on high multiplicity passage (data not shown). Taken together, our data indicate that normal replication of “non-defective” PIV5-VΔC does not activate the IFN-β promoter even though it does not encode a functional IFN antagonist. Rather, the induction of IFN by PIV5-VΔC in this system is primarily due to the presence of DI viruses.

## Discussion

A great deal of work has been done previously on the induction of IFN by paramyxoviruses and the role of their IFN antagonists in blocking activation of IFN induction pathways. Since these studies have focused on data obtained from cell population experiments, they failed to analyse the process of IFN induction in individual cells. We have previously examined the induction of IFN at the single-cell level (using our A549/pr(IFN-β).GFP cells) during infection with a range of wild-type negative-sense viruses, and observed heterocellular IFN-β promoter activity caused by differences in the infecting virus population (Chen et al., 2010). One possible explanation for this heterogeneity is that a virus infecting a cell that will go on to express GFP has been unable to block IFN induction by PAMPs that are generated during normal virus transcription or replication, either due to defective function or a loss of expression of the IFN antagonist. If the loss of a functional V protein were the primary reason for IFN induction in infected cells, then it would be expected that PIV5-VΔC would activate the IFN-β promoter in all infected cells. However, we found instead that only a small minority of A549/pr(IFN-β).GFP reporter cells expressed GFP when infected with our working stock of PIV5-VΔC. This was not due to differences in the ability of individual cells to respond to IFN inducers since the majority of cells expressed GFP when infected with a preparation of MuV known to be a good IFN inducer due to the presence of DI viruses. Similarly, by sequential high multiplicity passage of PIV5-VΔC to increase the number of DI viruses present, we could generate a PIV5-VΔC stock that activated GFP expression (and therefore the IFN-β promoter) in 80% of cells, consistent with previous studies indicating a correlation between paramyxovirus DI viruses and IFN induction (Johnston, 1981; Strahle et al., 2006). Moreover, the increase in GFP expression seen with our DI-rich PIV5-VΔC correlated with an increase in IRF3 phosphorylation, ISG expression and IFN secretion by infected cells, suggesting that our GFP reporter system faithfully represents the activation of the IFN response in infected cells. We also observed that the IFN-β promoter was only activated in a minority of cells within developing PIV5-VΔC plaques, and furthermore, plaques could be found in which no cells expressed GFP at all. Since the uninfected cells surrounding these GFP-negative plaques were also negative for the ISG product MxA, clearly no endogenous IFN-β had been secreted by any of the cells within these plaques, further indicating a close correlation between the activity of our reporter gene and the activity of the endogenous IFN-β gene.

The most striking result of this study was that the loss of the PIV5 IFN antagonist did not lead to IFN-β promoter activation in all PIV5-VΔC-infected cells. We considered the possibility that PIV5 may encode an uncharacterised trans-acting inhibitor of IFN-β induction that remains intact in the PIV5-VΔC genome and hence could limit the number of cells expressing GFP. However, in related studies we have found that whilst infection of cells with PIV5 wt blocks their ability to activate the IFN-β promoter in response to a variety of PAMPs, infection with PIV5-VΔC vM0 does not (data not shown). The data presented here challenge the notion that paramyxoviruses generate PAMPs capable of activating the IFN response during their normal replication cycle, and we suggest that these PAMPs are not generated during non-defective PIV5 transcription and replication. It is of note that the replication of negative strand RNA viruses is strictly linked to encapsidation of virus genomes and antigenomes (Gubbay et al., 2001), thereby hiding any potential structures in the genomes/antigenomes that could be recognised by RIG-I/MDA-5 (Goodbourn and Randall, 2009). Furthermore, it has been reported that PIV5 strictly regulates its transcription and replication processes in order to limit the production of aberrant RNAs that may otherwise activate the IFN response (Dillon and Parks, 2007; Gainey et al., 2008; Lin et al., 2005; Manuse and Parks, 2009). In addition to its role as an IFN antagonist, the V protein itself controls both PIV5 transcription and replication (Lin et al., 2005). In this regard it may be expected that due to the extensive deletion in V in PIV5-VΔC this V-dependent regulation would be altered, leading to a possible increase in the generation of PAMPs capable of inducing IFN. However, our data indicate that the loss of this fine control of transcription and replication does not affect the level of IFN induction during infection, since we do not see IFN-β promoter activation in the majority of PIV5-VΔC-infected cells.

Since we have shown that non-defective PIV5-VΔC does not generate PAMPs capable of activating the IFN-β promoter during its normal replication cycle, we suggest that, in this reporter system, DI viruses (which must be generated frequently during PIV5 replication, given the ease with which we were able to generate DI virus-rich stocks) are primarily responsible for IFN induction by PIV5. These results also raise the question of the role of V in blocking IFN induction if PAMPs are not produced/exposed during normal virus replication. One possibility is that V blocks IFN induction by PAMPs that are generated by DIs, either within cells in which the DI was initially generated, or in cells that have been co-infected with a wt virus and an IFN-inducing DI. Alternatively, infection of certain cells (e.g., plasmacytoid dendritic cells) may result in defective or altered virus replication and the generation of PAMPs capable of activating the IFN response, and it is the function of V to block IFN induction within these cells.

## Materials and methods

### Cells, viruses and IFN

Vero, A549 and 293 cells, and their derivatives, were grown as monolayers in Dulbecco's modified Eagle's medium (DMEM) supplemented with 10% foetal bovine serum at 37 °C. PIV5 (strain W3A; Choppin, 1964), PIV5-VΔC (He et al., 2002) and MuV(ori) (Enders; Chen et al., 2010) were grown and titrated under appropriate conditions in Vero cells or, where stated, in A549 cells. Virus infections were carried out in DMEM supplemented with 2% foetal bovine serum. The construction and properties of the A549/pr(IFN-β).GFP cell line have been previously and extensively described (Chen et al., 2010). IFN-α (Roferon A; Roche) was used at 1000 IU/ml.

### Generation of DI-rich virus preparations

To generate DI virus-rich stocks of PIV5-VΔC, Vero cells grown in 75 cm^2^ flasks were infected at an MOI of 5 PFU/cell with our working stocks (vM0). Every 2 – 3 days the culture medium was harvested; half was frozen at − 70 °C for subsequent analysis and the other half used to infect the next 75 cm^2^ flask. Sequential preparations of these stocks are referred to as vM1, vM2, etc.

### Analysis of copyback RNA in DI-rich virus preparations

Total cellular RNA was prepared from 293 cells infected with PIV5-VΔC vM0 or vM2 stocks, prepared as described above, at an MOI of 5 PFU/cell. Two micrograms of total cellular RNA was reverse transcribed with RevertAID reverse transcriptase (Fermentas) using oligos A (5′-CCAAGAAGACCTAAATTGTAAGGAG-3′) or B (5′-CTCCTTACAATTTAGGTCTTCTTGG-3′) which are complementary to the (+) and (−) strand, respectively, for nucleotides 14760–14784. cDNA was subsequently amplified with AccuPrime Pfx DNA polymerase (Invitrogen) using either oligo A paired with oligo C (5′-GGATTGGATCCGAATGCTGCCAAGGGGAAAACCAAGATTAATCCTCT-3′) or oligo B paired with oligo C (oligo C is complementary to nucleotides 15218–15246, the terminus of the PIV5 genome). Amplification products were separated by agarose gel electrophoresis, recovered and cloned as blunt-ended fragments into pJET1.2 (Fermentas) and sequenced (GATC Biotech).

### Immunofluorescence, immunoblotting and FACS analysis

The procedures for immunoblotting and immunofluorescence have previously been described (Carlos et al., 2005). Antibodies used in these procedures included monoclonal antibodies against PIV5 NP (PIV5-NPa; Randall et al., 1987), PIV5 V (raised against the C-terminus; a kind gift from R. Lamb), phospho-IRF3 (Cell Signaling Technology), GFP (Roche), and β-actin (Sigma). Polyclonal antibodies used included those raised against ISG56 (Santa Cruz) and MxA (Santa Cruz). Immunofluorescence was examined with either a Nikon Microphot-FXA immunofluorescence microscope or a Zeiss LSM 5 Exciter confocal microscope. For FACS analysis, A549/pr(IFN-β).GFP cells were trypsinised to obtain single-cell suspension and fixed in PBS/5% formaldehyde. GFP expression was examined using a BD FACScan flow cytometer.

### Interferon assays

The amount of IFN secreted by cells was determined by a CPE-reduction bio-assay. Briefly, culture supernatants from infected cells were harvested, centrifuged at 1500 × *g* for 10 min to pellet cellular debris, UV-treated to inactivate residual virus, then serially diluted 2-fold and added to A549/BVDV-N^pro^ cell monolayers (Hilton et al., 2006) for 18 h prior to infection with EMCV (0.05 PFU/cell). Monolayers were fixed 2–3 days post-infection (with PBS/5% formaldehyde) and cytopathic effect (CPE) was monitored by staining with 0.1% crystal violet.
